# Microwave-assisted organic acids and green hydrogen production during mixed culture fermentation

**DOI:** 10.1186/s13068-024-02573-7

**Published:** 2024-09-28

**Authors:** Maximilian Barth, Magdalena Werner, Pascal Otto, Benjamin Richwien, Samira Bahramsari, Maximilian Krause, Benjamin Schwan, Christian Abendroth

**Affiliations:** 1 Institute for Waste Management and Circular Economy, TUD Dresden University of Technology, Pirna, Germany; 2 Robert Boyle Institute e.V, Jena, Germany; 3 Dresden-concept Genome Center, CMCB Center for Molecular and Cellular Bioengineering, TUD Dresden University of Technology, Dresden, Germany; 4 Department of Circular Economy, Brandenburg University of Technology Cottbus-Senftenberg, Cottbus, Germany

**Keywords:** Acidification, Hydrogen production, Volatile fatty acids production, Microwave heat shocks, 16-S-rRNA sequencing, Dark fermentation

## Abstract

**Background:**

The integration of anaerobic digestion into bio-based industries can create synergies that help render anaerobic digestion self-sustaining. Two-stage digesters with separate acidification stages allow for the production of green hydrogen and short-chain fatty acids, which are promising industrial products. Heat shocks can be used to foster the production of these products, the practical applicability of this treatment is often not addressed sufficiently, and the presented work therefore aims to close this gap.

**Methods:**

Batch experiments were conducted in 5 L double-walled tank reactors incubated at 37 °C. Short microwave heat shocks of 25 min duration and exposure times of 5–10 min at 80 °C were performed and compared to oven heat shocks. Pairwise experimental group differences for gas production and chemical parameters were determined using ANOVA and post–hoc tests. High-throughput 16S rRNA gene amplicon sequencing was performed to analyse taxonomic profiles.

**Results:**

After heat–shocking the entire seed sludge, the highest hydrogen productivity was observed at a substrate load of 50 g/l with 1.09 mol H_2_/mol hexose. With 1.01 mol H_2_/mol hexose, microwave-assisted treatment was not significantly different from oven-based treatments. This study emphasised the better repeatability of heat shocks with microwave-assisted experiments, revealing low variation coefficients averaging 29%. The pre-treatment with microwaves results in a high predictability and a stronger microbial community shift to *Clostridia* compared to the treatment with the oven. The pre-treatment of heat shocks supported the formation of butyric acid up to 10.8 g/l on average, with a peak of 24.01 g/l at a butyric/acetic acid ratio of 2.0.

**Conclusion:**

The results support the suitability of using heat shock for the entire seed sludge rather than just a small inoculum, making the process more relevant for industrial applications. The performed microwave-based treatment has proven to be a promising alternative to oven-based treatments, which ultimately may facilitate their implementation into industrial systems. This approach becomes economically sustainable with high-temperature heat pumps with a coefficient of performance (COP) of 4.3.

**Graphical abstract:**

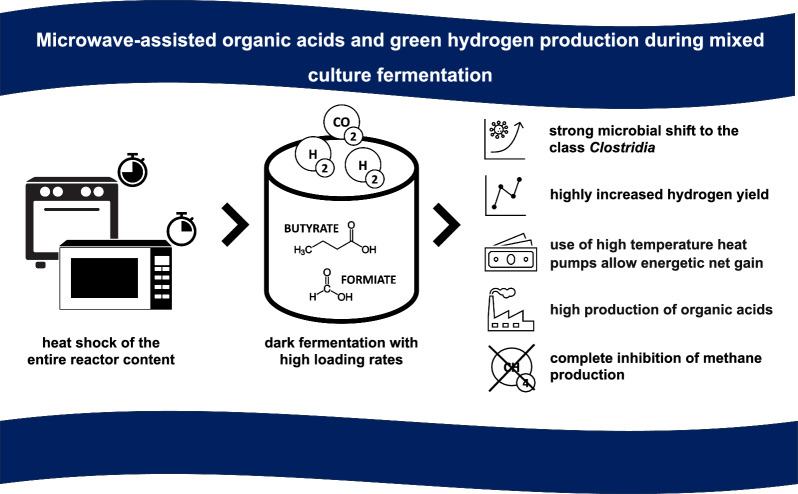

**Supplementary Information:**

The online version contains supplementary material available at 10.1186/s13068-024-02573-7.

## Background

Anaerobic digestion (AD) is a technology that enables the production of methane from various organic resources and residues, such as straw and corn stover [1], chicken manure [2], and food waste [3]. AD is not only a treatment technology but also a potential node in biorefinery. Existing industrial AD plants already generate biogas from biodegradable waste. Biogas can be upgraded to biomethane, which has similar qualities to natural gas. AD facilitates efficient waste biodegradation by harnessing diverse microbial communities to break down complex organic matter under oxygen-depleted conditions [4]. This biodegradation process not only reduces the volume and mass of the biowaste but also stabilises it, minimising potential environmental pollution. However, AD-faces significant challenges when compared to established technologies that utilise fossil fuels. In Germany, the monetary shortcoming for AD plants is counterbalanced by the German Renewable Energy Sources Act [5]. Therefore, technological and conceptual advancements are required to make AD a self-sustained technology. Potential means of achieving this is to intertwine AD with existing bio-based industries to create synergetic effects. For instance, Sawatdeenarunat et al. discussed the possibility of producing valuable side products such as syngas, methanol and butanol, [6] from extracted solids, the digestate, or the biogas itself. A particularly promising approach to producing valuable side products is the application of two-stage anaerobic digestion technology with a separate acidification stage. Acidogenic microorganisms cultivated under high substrate concentrations and low pH conditions facilitate the accumulation of short-chain fatty acids (SCFAs; C2–C5). Anaerobic fermentation pathways, including butyric acid fermentation, propionic acid fermentation, and mixed-acid fermentation, are known to predominantly generate short-chain fatty acids (SCFAs) [7–10]. Due to various applications in chemical industries, these SCFAs are often referred to as platform or starting chemicals [11]. In the presence of electron donors, such as ethanol or lactate, SCFAs could even be further valorised due to a mechanism known as chain elongation, which yields caproic acid, a valuable medium-chain fatty acid [12]. Another promising metabolite formed during AD is hydrogen [13]. A two-stage digestion process is well suited for microbial hydrogen production [14]. Using acidic pre-treatment stages of anaerobic digesters for hydrogen production is a process that is very similar to dark fermentation. Several studies have used chemical treatment [15], aeration, and electrical treatments [16] to stimulate hydrogen production. Dark fermentation is an approach to yield hydrogen from mixed culture fermentation with high substrate concentrations, which has been described extensively in the literature [17–19].

AD involves around 300 operational taxonomic units, which cover 80% of all reads from 16S rRNA gene amplicon high-throughput sequencing of samples for 32 full-scale digester plants [20]. Such high diversity makes it difficult to achieve a stable microbial community that can yield a predictable hydrogen output. Another problem that occurs when coupling a dark fermentation stage with a methanogenic reactor is the contamination with methanogens. By recycling the liquid phase from the methane stage to the acidification stage, methanogens could contaminate the acidification stage. Although high organic loading rates (OLRs) and low pH levels tend to suppress methanogenesis [21, 22], the formation of methane during dark fermentation has been described before [23]. Interestingly, heat shocks can help optimise this problem. Although methanogenesis is not fully suppressed, it has been indicated recently that heat shocks can reduce the amount of methane produced in separated acidification stages [24]. The fact that heat shocks can simultaneously suppress methanogenesis and contribute to an enrichment of hydrogen-forming bacteria makes them particularly interesting. Although several research articles already address the application of heat shocks on the inoculum to improve hydrogen formation, the authors of the present work have identified few scientific articles on experiments where the whole substrate and medium were heat–shocked. For industrial applications, treatment of the complete seed sludge would be more practical. The presented work closes this research gap by investigating the industrial suitability of heat–shocks for separated acidification stages via heating up the entire seed sludge.

The utilisation of existing waste streams in the form of various fermentation residues from biogas production offers new cascading recycling stages through heat-shock pre-treatment. In the present study, the application of microwaves was assessed in comparison to heat shocks in the oven for the first time. Furthermore, the effect of heat shocks on the underlying microbial community and the co-production of volatile fatty acids in the acidification stage were investigated. Shorter and therefore more economical heat shocks due to microwaves could represent another step towards integrating dark fermentation and methane production. In the future, this could help transform existing anaerobic digesters into multistage, multiproduct biorefinery plants.

## Methods

### Experimental set-up

**Fig. 1:**
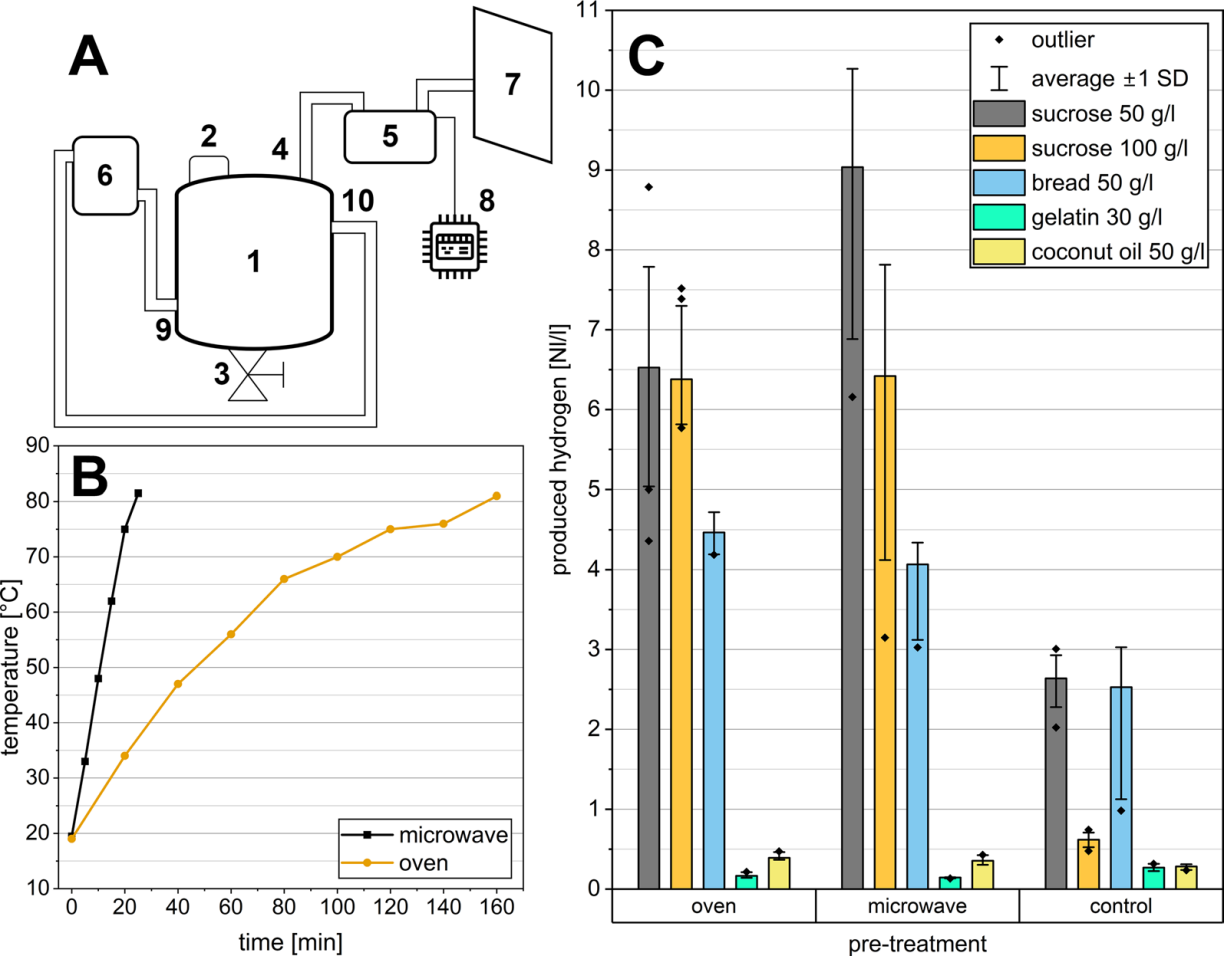
**A** Experimental set-up: double-walled reactors (1) with a volume of 5 l each. Each reactor was filled through an input port (2), and acidified hydrolysate was removed through a port at the bottom (3). For heating, a water pipe was connected to the input (9) and output (10) ports of the double-wall clearance. Water was heated using a thermostat (6). The gas produced was measured by a MilliGascounter (5), connected to a tube (4), and stored in a gas bag (7). The time-resolved gas production was recorded with an Arduino (8) connected to the gas counter. **B** Average incubation time for heat shock up to a minimum of 80 °C for pre-treatment with oven (set to 130 °C) and microwave (set to 1000 W). **C** Substrate variation tests: Comparison of hydrogen production with sucrose, mixed rye bread, gelatine and coconut oil as feeding substrates and sewage sludge as inoculum. Both control and heat shock (oven and microwave) scenarios were tested

Six identical double-walled tank reactors (3.3 l working volume) from the company Lehmann-UMT GmbH (Germany) were applied (Fig. 1A). The batch experiments were fed with different substrate concentrations of 50, 75 and 100 g/l of sucrose. To enable hydrogen production during mixed-culture fermentation, high substrate to inoculum (S/I) ratios were applied. Similar S/I ratios were applied in other studies, such as by [25], who applied high organic loading rates (OLRs) of up to 160 g COD/l/*d. Notably, previous studies have typically utilised considerably lower substrate concentrations; for example, [26] applied just 50 g/l sucrose. In the present study, all reactors were operated at 37 ± 1 °C. Generally, the experimental procedure can be divided into the following steps: (1) fermentations to distinguish the impact of heat shocks on hydrogen formation for proteins, fat, and carbohydrates; (2) comparison of microwave and oven-based heat shocks as pre-treatment to enhance hydrogen formation; (3) kinetic analysis of the different experimental scenarios; and (4) microbial sampling and 16 s sequencing.

The entire digestion mixture was subjected to heat shocks before being filled into the reactor. Different methods of heating were tested. For the heat shock, the slurry was heated using both oven and microwave. Pre-treatment of the inoculum using oven-generated heat shock within the range of 80 °C to 200 °C has been extensively documented in scientific literature [27–30]. In the present study, the sludge was incubated using a laboratory oven (Binder ED 115) at 130 °C in 1 L Schott flasks for up to 3 h. Using a microwave (Panasonic NN-ST45KW), the inoculum sludge was heated in 5 L measuring cups at 1000 W for a considerably shorter time of just 25 min. Both methods were used to heat the sludge up to 80 °C–90 °C to eliminate hydrogen-consuming microorganisms. Figure 1B presents the incubation times and heating curves. After the heat shock, the sludge was cooled down to 40 °C. Mohanakrishna and Pengadeth reported that heat shocks are often performed in the range of 65 °C–121 °C with exposure times between 1 to 10 h [31]. In the present work, the temperature of the heat shock is up to 80 °C ± 1 °C, while the exposure times are only 5 min–10 min for the microwave and 10 min–20 min for the oven pre-treatment. These lower and shorter conditions were chosen based on the experiments of Wong et al. [32] and aimed to reduce the heat shock duration and exposure time even further. In their experiments, Wong et al. demonstrated that effective heat shock pre-treatment of anaerobic sludge could be achieved at lower temperatures (65 °C–85 °C) and for 45 min–60 min. Lower heat shock temperatures and shorter durations were chosen for their higher energy efficiency. In this regard, [33] suggest that methanogen suppression might be achievable with milder heat shock conditions. In the present study, the fermentation broth was continuously stirred to ensure homogenous temperature distribution during the heat shock pre-treatment.

The experiments tested two conditions: substrate concentration and the heat-shock method. Three feeding scenarios (50, 75, 100 g/l) were combined with three treatment methods (control, microwave, oven). To ensure high statistical confidence, each scenario was repeated at least six times, resulting in a total of 69 data points analysed. The data were composed of 29 control experiments, 20 oven-pretreated experiments, and 20 microwave-pre-treated experiments.

### Inoculum and substrate

Dark fermentation experiments were performed using digested sewage sludge as seed sludge. In addition to carbohydrates, substrates that are rich in proteins and fats were tested in a first set of experiments to evaluate their suitability in a heat shock context (Fig. 1C). Based on its better performance, sucrose was chosen as the carbon source for the ongoing experiments. The sewage sludge was retrieved from the digester of a municipal wastewater treatment plant in Saxony, Germany. Table 1 lists the working parameters of the wastewater treatment plant.

**Table 1:** Working parameters and digested sludge parameters of the wastewater treatment plant in Saxony

Parameter	Value
Temperature	30–35 °C
Solids retention time (SRT)	16.5 days
pH	7.6
Total solid content (TS)	2.8%
Volatile solids (VS)	57.9%
Ammonium content (NH_4_-N)	1109 mg/l
Total organic carbon (TOC)	336.7 mg/g
Soluble chemical oxygen demand (COD)	1220 mg/l
Dissolved organic carbon (DOC)	329.9 mg/l
C:N ratio*	0.2 mg/l
Total volatile fatty acids (TVFA)	27.1 mg/l
Lactic acid (C_3_H_6_O_3_)	–
Formic acid (CH_2_O_2_)	4.5 mg/l
Acetic acid (CH_3_COOH)	16.4 mg/l
Propionic acid (C_3_H_6_O_2_)	1.9 mg/l
Iso-butyric acid (C_4_H_8_O_2_)	0.8 mg/l
Butyric acid (C_4_H_8_O_2_)	3.5 mg/l
Valeric acid (C_5_H_10_O_2_)	–
Calcium (Ca)	118.5 mg/l
Magnesium (Mg)	33.7 mg/l
Potassium (K)	178.3 mg/l
Sodium (Na)	16.5 mg/l
Copper (Cu)	–
Zinc (Zn)	160.3 mg/l
Nickel (Ni)	53 mg/l
Molybdenum (Mo)	–
Manganese (Mn)	54.5 mg/l

^*^C: N ratio of the liquid phase was determined using the DOC: TKN ratio

### Analytical methods

#### Gas volume and composition

The gas composition, consisting of hydrogen (H_2_), carbon dioxide (CO_2_), and methane (CH_4_), was determined using a BlueVary gas analyser from BlueSens (Herten, Germany). To ensure anaerobic conditions, the oxygen (O_2_) and hydrogen sulphide (H_2_S) contents were measured using the X-AM 8000 gas detector by Dräger (Lübeck, Germany).

The amount of hydrogen sulphide was very low (usually a few ppm) and was therefore omitted in the later results. To calculate the overall yield, the gas contained in the headspace was considered as well. The yield of hydrogen was estimated using Eq. 1, which considers the density and molar mass of hydrogen, the input mass and molar mass of the substrate (sucrose), and the number of hexose molecules per molecule of sucrose. The equation assumes that the substrate is completely consumed.

$$\text{y}= \frac{V(H_{2})\times  \rho (H_{2})\times M(Sucrose)}{M(H_{2})\times m(Sucrose) \times N(Sucrose)}$$

y is the yield [mol H_2_/mol hexose]. V(H_2_) represents the generated volume of hydrogen [Nl]. ρ(H_2_) represents the density of hydrogen [g H_2_/Nl]; (0.0899 g H_2_/Nl). M(Sucrose) is the molar mass of sucrose [g Suc/mol Suc]; (342.3 g Suc/mol Suc). M(H_2_) is the molar mass of hydrogen [g H_2_/mol H_2_]; (2.01588 g H_2_/mol H_2_). m(Sucrose) is the mass of sucrose [g Suc/l Input]. N(Sucrose) is the number of hexose molecules per sucrose molecule; (1.9).

#### Digestate analysis

For chemical analysis of the digestate, 300 ml of samples of the initial sludge was taken before heat shock, along with samples from each reactor after a complete fermentation phase (7 days). For the metagenomic analysis, additional samples of the digestate were taken at the end of the fermentation process, which corresponded to an incubation period of 7 days. The solubilised COD was measured in the liquid phase after centrifugation at 1200 relative centrifugal force (RCF) and subsequent vacuum filtration through a 0.2 µm cellulose-acetate filter (Sartorius AG, Göttingen, Germany). The COD was determined using the Spectroquant COD kit (VWR, Germany) according to the manufacturer’s guidelines. The spectrum of VFAs including lactic acid, formic acid, acetic acid, propionic acid, iso-butyric acid, butyric acid and valeric acid was determined via ion chromatography (IC). A cation exchange column (Metrosep Organic Acids 250/7.8 column; Model: 882 Compact IC plus, Metrohm AG, Herisau, Switzerland) was used for this purpose. The mobile phase had a concentration of 0.6 mmol/l of perchloric acid and 10 mmol/l of lithium chloride. The detection limit was 0.25 mg/l. TVFAs were analysed as the sum of all detected VFAs. Initially, heavy metals were measured using microwave digestion and inductively coupled plasma (ICP) (PerkinElmer Optical-Emission Spectrometer Optima 8000/ S10 Autosampler). Nitrogen contents including total Kjeldahl nitrogen (TKN) and NH_4_-N, were determined according to ISO 5663. Real-time pH monitoring was not employed during the main experiments to avoid potential disruption of gas production. The pH was measured at the beginning and end of each fermentation process.

### Metagenomics analysis of the microbial community

For microbial community analysis with 16S rRNA gene amplicon high-throughput sequencing, an aliquot of 3 ml of each sample stored in ethanol was centrifuged and washed with sterile phosphate-buffered saline (PBS) until the supernatant was clear. DNA was extracted from the resulting pellets using a Qiagen DNeasy PowerSoil Kit. DNA was quantified using the Qubit 1 × dsDNA Kit from ThermoFisher. The extracted metagenomic DNA was used to amplify the hypervariable V3–V4 region of the 16S ribosomal RNA gene. The conserved regions V3 and V4 (470 bp) of the 16S rRNA gene were amplified using PCR cycling, with initial denaturation at 95 °C for 3 min; 25 amplification cycles (20 s at 98 °C, 15 s at 60 °C, 30 s at 72 °C); and 5 min extension at 72 °C. The following primers were used:

- 341F (5′ ACACTCTTCCCTACACGACGCTCTTCCGATCT NNNNN CCTAYGGGRBGCASCAG 3′)
- 806R (5′ GTGACTGGAGTTCAGACGTGTGCTCTTCCGATCT GGACTACNNGGGTATCTAAT 3′)

Amplification was performed using the Thermo Scientific Phusion High-Fidelity DNA polymerase kit. The purification of the amplicons was carried out after each PCR with the 1 × v/v AmPure XP beads from Beckmann Coulter. The purified 16S amplicons were indexed with a second PCR containing TruSeq Illumina Indexing Primers (cycling conditions 98 °C 30 s denaturation, 98 °C 15 s, 65 °C 75 s, 10 cycles, final elongation 5 min 65 °C, 4 °C overnight). Final libraries were again purified with 1 × v/v AmPure XP beads and quality-assessed on an Agilent FragmentAnalyzer (1-6000 bp NGS Kit). Resulting libraries were equimolarly pooled and sequenced. Sequencing was performed using 2X250pb or 2 × 300pb paired-end cycle runs on an Illumina MiSeq instrument. The Illumina raw sequences were loaded into Qiime2 (v. 2021.2.0) [34]. The quality of the sequences was checked with the Demux plugin and the DA-DA2 pipeline integrated in Qiime2. It was used to trim and join the sequences, remove chimaeras and detect amplicon sequence variants (ASVs; > 99.9% similarity). The taxonomy of each sequence variant was determined using SILVA [35] as the reference database for taxonomic assignment.

### Statistical analysis

For the basic statistical evaluation of the test series, where each series included at least six measured values, the median was selected to better represent the tendency of the individual test results. To test the experimental groups for statistically significant group differences, the Levene test was first used as a test procedure for homogeneity of variance. Compared to the Bartlett test, the Levene test is significantly more robust when populations are not normally distributed [36]. For experimental groups that did not have homogeneity of variance, a WELCH ANOVA was performed [36, 37]. The groups with homogeneity of variance were compared using classic ANOVA. Pairwise experimental group differences were determined using post–hoc tests. The Tukey–Kramer test [38, 39] was used for this purpose, while the Games–Howell test was used for groups without homogeneity of variance [40]. For all tests, the significance level was set at α = 0.05.

## Results

### Dark mixed-culture fermentation with different substrates

**Fig. 2:**
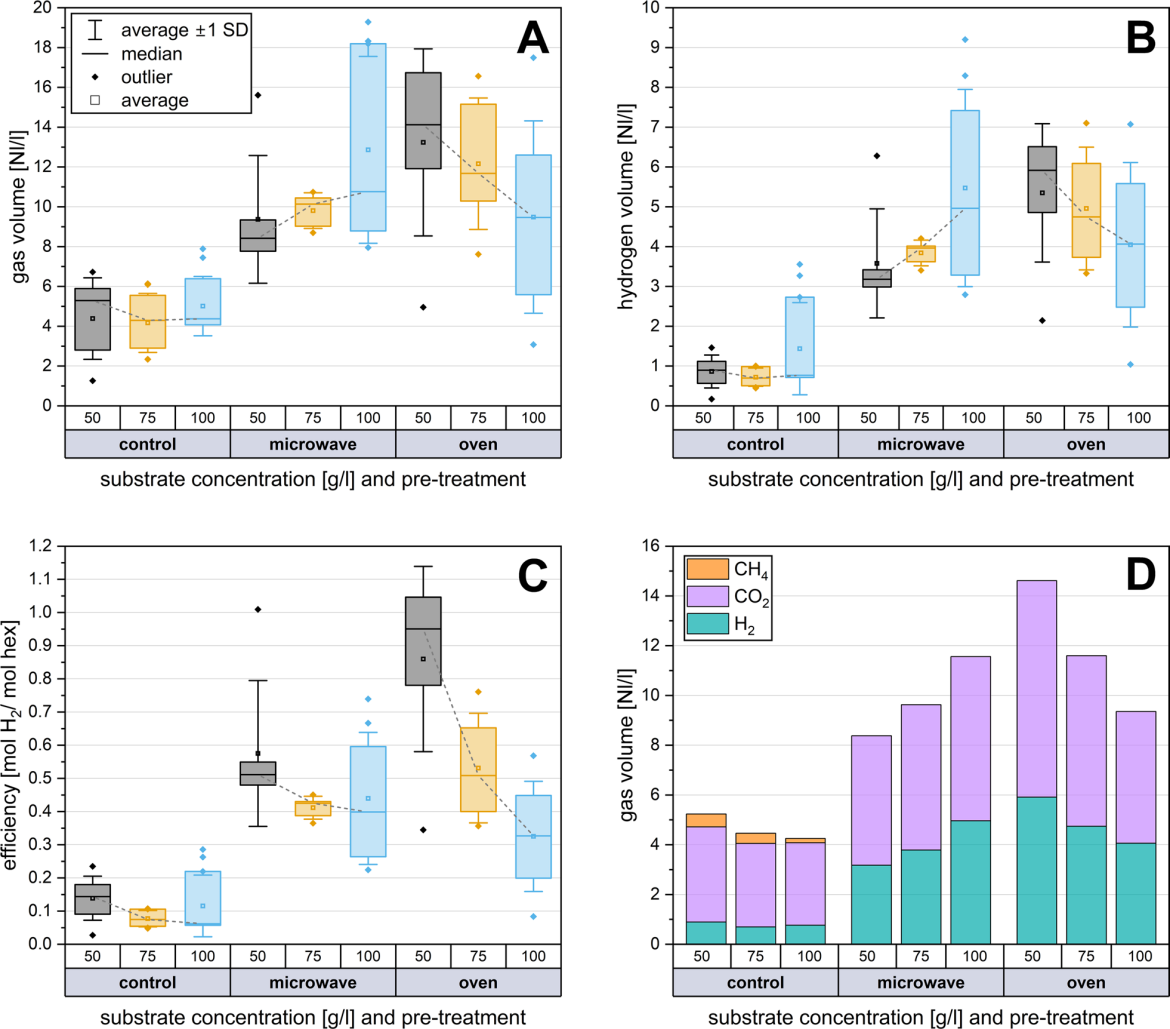
Application of heat shocks to optimise hydrogen productivity: different concentrations of substrate (sucrose) and different pre-treatments (oven and microwave) were compared. **A** Maximal gas productivity and **B** the highest hydrogen production were reached with a substrate concentration of 50 g/l and pretreated with oven heat shock. All fermentations were performed at least 5 times. **C** The highest hydrogen efficiency was achieved with 50 g/l sucrose in both microwave and oven experiments. **D** Gas composition depending on the feeding amount and pre-treatment. The median values were linked to a trend line to better visualise the effects of the different substrate concentrations

Hydrogen production for the fatty substrates resulted in generally low production rates, with maximums of 0.32 Nl/l (normalised litre gas per litre working volume) for gelatine and 0.47 Nl/l for coconut oil. Protein-rich mixed rye bread produced a maximum hydrogen yield of 4.46 Nl/l with oven pre-treatment and 4.07 Nl/l with microwave pre-treatment. However, this was lower than the results obtained with 50 g/l and 100 g/l sucrose. The respective hydrogen yields are shown in Fig. 1C. In the control group without treatment, a hydrogen yield of only 0.62 Nl/l was achieved when loaded with 100 g/l sucrose. In the first set of experiments, proteins (gelatine) and lipids (coconut oil) were barely metabolised into biogas (production of 0.27 and 0.28 Nl/l, respectively, Fig. 2). Due to this negligible biogas formation, no controls without substrates were regarded as necessary. In contrast, both heat shock scenarios resulted in a hydrogen production of 6.42 Nl/l at this high feeding overload. The highest hydrogen production was achieved with 50 g/l sucrose and oven-heated pre-treatment of the sludge. Overall, the heat-treated trials produced twice as much total gas compared to the untreated control.

### Combination of heat shock pre-treatment and high feeding rates

As shown in Fig. 1C, fats and proteins could not match the hydrogen production rates of sugar in dark fermentation combined with heat shock. Therefore, subsequent experiments were focused on very high feeding rates of sucrose as a substrate. As presented in Fig. 2, different sucrose concentrations (50, 75 and 100 g/l) were compared in terms of hydrogen production, to determine the optimal substrate concentration. Assuming an idealised uniform consumption of the substrate, this would correspond to loading rates of 7, 11 and 14 g COD/l·d. The highest average hydrogen yields in mol H_2_ per mol hexose were obtained within the heat-shock-treated experiments, with a loading of 50 g/l sucrose (Fig. 2C). Microwave- and oven-pretreated sludges yielded hydrogen formation efficiencies of 0.51 and 0.95 mol H_2_/mol hexose, respectively. The peak values for the hydrogen efficiencies were measured at 1.01 (microwave) and 1.09 mol H_2_/mol hexose (oven), both with a substrate concentration of 50 g/l sucrose.

**Fig. 3:**
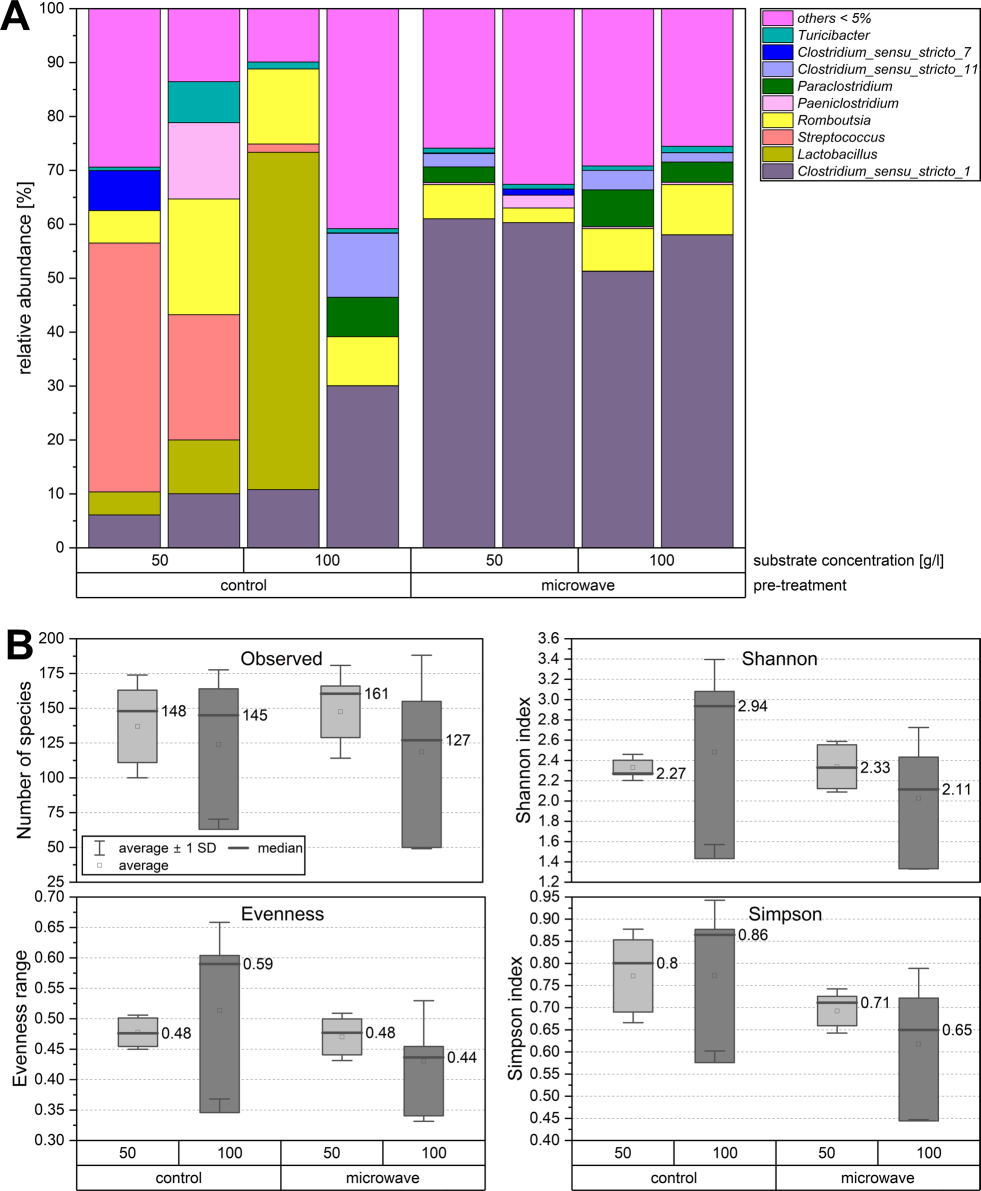
**A** Microbial community on genus level as relative abundances differentiated according to pre-treatment and sucrose concentration. Only the most abundant microorganisms with an abundance > 5% are presented; others with abundances below 5% are summarised as others < 5%. Duplicates were sequenced for each condition. **B** Alpha Diversity of the microbial community for genus level data of microwave and control samples, substrate concentrations of 50 and 100 g/l of sucrose

The untreated control showed the highest median hydrogen formation at 100 g/l sucrose with 0.2 mol H_2_/mol hexose, corresponding to 0.77 l hydrogen per litre of working volume. In the heat-treated experiments, the lowest median hydrogen yield was produced with 100 g/l sucrose, with 0.39 and 0.33 mol H_2_/mol hexose for microwave and oven pre-treatment. As depicted in Fig. 3A and B, increasing the feeding rates led to a rise in both the total volume and the absolute quantity of hydrogen, but only in the experiments that underwent microwave pre-treatment. Conversely, in the oven-treated experiments, the volumes decreased, leading to an inverse effect. Consequently, the efficiency of hydrogen formation in the oven experiments dropped significantly by approximately two thirds from 50 g/l to 100 g/l. However, the decrease in hydrogen yield with microwave pre-treatment was only 28%. In the untreated control experiments, there was only a slight change in gas volumes.

Figure 2A illustrates that the heat shocks significantly increased both the total gas volume and absolute hydrogen production (Fig. 2B) and improved hydrogen formation efficiency (Fig. 2C) compared to the untreated control experiments. In the heat shock trials (both oven and microwave), methane production was successfully eliminated (Fig. 2D).

As a result of the heat treatments, the relative standard deviation of the hydrogenation efficiency increased by 49% on average in the microwave pre-treatment and by 70% in the oven pre-treatment. The standard deviation of the oven tests was on average 32% higher than in the microwave tests. Pre-treatment with the oven was overall related to higher gas production compared to the microwave. However, the boxplots of the series of experiments reveal that the microwave shocks reduced the scatter of the results, an effect that was not observed in the experiments with oven treatment.

Distinct observations were made at a substrate concentration of 50 g/l sucrose. Here, the oven demonstrated a significant increase in absolute hydrogen production and consequently in hydrogen formation efficiency (Fig. 2B and C). A median of 5.92 l H_2_/l sludge was produced, corresponding to a hydrogen yield of 0.95 mol H_2_/mol hexose. For both heat shock methods, the median hydrogen formation efficiency shows a decreasing trend as the substrate concentration increases. This pattern is reflected in the total volume and hydrogen volume for the oven-based heat shock experiments (50 > 75 > 100 g/l with 0.95 > 0.51 > 0.33 mol H_2_/ mol hex). In contrast, specific hydrogen production increases with increasing feeding rates in the microwave pre-treatment experiments (Fig. 2C). Concurrently, the microwave-assisted hydrogen production efficiency remains more constant, decreasing by only 22% from 50 g/l to 100 g/l (65% decrease for oven pre-treatment). The distribution of gas volume indicates that in all experiments involving heat shock pre-treatment, the total hydrogen content in the gas increased to approximately 40% for microwave and 41% for oven-pretreated experiments. This is a significant increase when compared to the median of the control at 18% H_2_ (Fig. 2D). While in microwave-treated approaches the relative hydrogen fraction increased slightly from 38 to 42%, it remained constant at 40% to 42% in the oven-treated experiments. In both heat shock methods, the highest relative hydrogen content was obtained in the experiments with 100 g/l sucrose (microwave treatment: 43%; oven treatment: 43.5%). Methane was not produced in any of the heat-treated trials. The residual portion of the gas mixture consisted entirely of carbon dioxide.

**Fig. 4:**
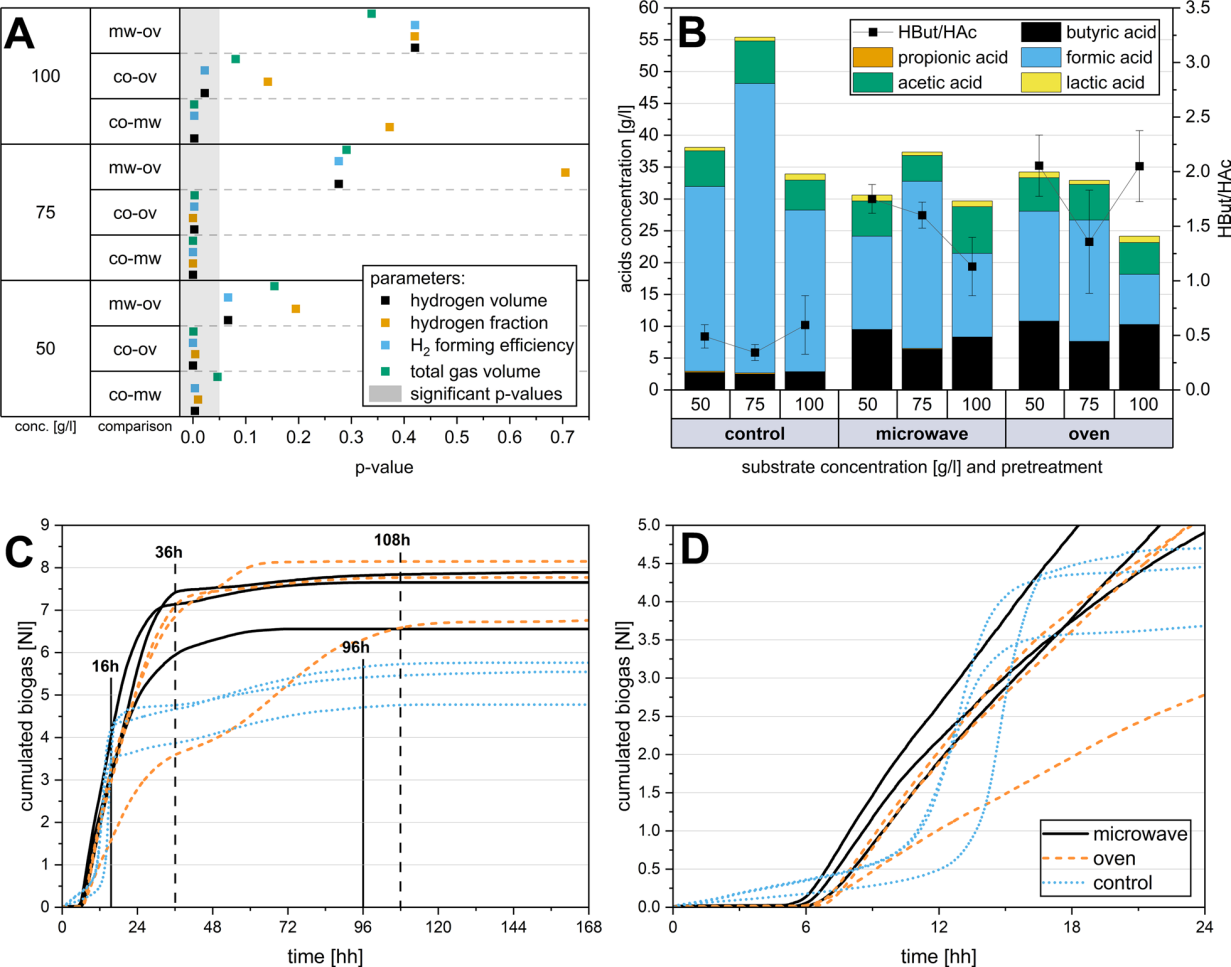
**A** Graphical representation of statistical significance (significant for *p* < 0.05) of group comparisons for hydrogen and total gas volumes, hydrogen formation efficiency and hydrogen fraction. The marker in the grey area indicates a significant difference between the respective comparison groups. **B** Production of volatile fatty acids (median) differentiated by treatment method and feeding amount. Volatile fatty acids were analysed in triplicate in all cases. **C** Course of gas production over seven days and **D** first 24 h for untreated (control) and heat-shock-treated (oven and microwave) experiments. Performed with sewage sludge and 75 g/l sucrose as feeding substrate

In general, thermal pre-treatment increased the hydrogen production capacity of anaerobic sludge in the dark fermentation experiments by at least a factor of 3.6 for the microwave and a factor of 5.3 for the oven compared to untreated experiments. The maximum increase of 6.8 times was obtained in oven-treated trials at 75 g/l substrate concentration and in microwave-assisted trials by 6.5 times at 100 g/l substrate concentration (microwave: 50 g/l–3.6 times, 75 g/l–5.4 times; oven: 50 g/l–6.6 times, 100 g/l–5.3 times). The strongest increase of 1.9-fold in the oven-treated trials over experiments with microwave treatment was achieved at a substrate concentration of 50 g/l. However, this did not result in statistically significant differences (*p* < 0.05) between microwave and oven pre-treatment in terms of overall hydrogen volume (p-values for all substrate concentrations in Fig. 4A). At 100 g/l sucrose concentration, no significant group differences between the control and oven for total gas volume (*p* = 0.08) and hydrogen content (*p* = 0.14) could be determined. For both the 50 g/l and 75 g/l substrate concentration loading rate experiments, there were statistically significant differences (*p* < 0.05) between both heat shock scenarios and the control for the parameters hydrogen volume, hydrogen fraction, hydrogen forming efficiency, and total gas volume.

### Microbial community taxonomy

The effect of microwave heat shock on the taxonomic profile and diversity of the microbial community was determined using 16S rRNA gene amplicon high-throughput sequencing. To capture the range of microbial community changes, the edges of the test spectrum were defined and analysed at 50 g/l and 100 g/l sucrose. The purpose of evaluating the microbial community at a substrate concentration of 50 g/l was to reflect the taxonomic shifts as a result of overfeeding less strongly. Microbial community analysis of the inoculum sludge, already previously performed by [41] for the same industrial sewage sludge digester in Dresden (Germany), revealed a predominance of *Bacteroidetes, Bacillota, Patescibacteria, Chloroflexi, Proteobacteria, Cloacimonetes, Verrucomicrobia,* and *Spirochaetes* at the phylum level. They also identified *Rikenellaceae_DMER64, Candidatus Cloacimonas, Sedimentibacter, Smithella*, and *Cloacimonadaceae* as the most abundant genera within the sludge. Our experiments revealed a significant decrease in the relative abundance of *Bacteroidota* and *Patescibacteria* compared to the initial microbial community. Conversely, the proportion of *Bacillota* increased by approximately ninefold. Notably, unlike [24], an very low abundance of *Proteobacteria* was detected within the heat-resistant microbial community. In the present study microwave treatment effectively eliminated non-spore formers and homogenised the microbial community towards the class *Clostridia* in experiments with 50 g/l substrate concentration. In experiments with 100 g/l overfeeding, a similar effect was observed but with slightly lower abundances of *Clostridia*. With a substrate concentration of 50 g/l sucrose, *Clostridia* predominantly colonised the microwave-treated substrates at an average abundance of 81% (100 g/l, 79%). As depicted in Fig. 3A, the microbial growth community in the microwave-treated reactions was composed of 51%–61% *Clostridium *sensu stricto* 1*. In contrast, the genera *Streptococcus* and *Lactobacillus* dominated the control experiments, making up 23% to 46% and 63% of the total microbial community. The heat shock succeeded in completely suppressing *Lactobacillus*, a lactic acid producer. Larger amounts of the genus *Romboutsia* were found in all scenarios with an average abundance of 10%. Larger amounts of the genus *Paraclostridium* were found in highly overfed experiments with abundances of only 4%–7%. Figure S01 in the supplementary section shows the very diverse group of all identified microorganisms with abundances below 5%. Microwave heat shock pre-treatment not consistently resulted in a reduction of observed species diversity. For a substrate concentration of 100 g/l the number of observed genera was reduced by an average of 20% compared to the control, (see Fig. 3B). While the Shannon index showed no significant difference in diversity for 50 g/l, the Simpson index indicated a reduction in species diversity for both substrate concentrations in the microwave-treated trials. Control scenarios exhibited a higher evenness, while microwave-treated microbial communities pointed to a highly uneven distribution of species. The strong shift towards *Clostridium *sensu stricto* 1* and Clostridia in general in the heat-treated experiments led to a more uneven distribution of species. The experiments indicated that the combination of microwave heat–shocks and high substrate concentrations of 100 g/l has intensified this effect compared to substrate concentrations of 50 g/l. This generally low equal distribution is also reflected in the taxonomic profile, which indicates that an average of 28% of the microbial community consists of smaller groups of microorganisms with less than 5% relative abundance (Fig. 3A).

### Production of volatile fatty acids

In general, TVFA production was approximately 30 to 40 g/l in heat-shocked experiments and between 35 and 55 g/l in non-pretreated experiments as a result of the higher formic acid production. The thermally pretreated experiments demonstrated a significant shift in the produced acids towards butyric acid. In heat-shocked experiments, the molar ratio of butyric acid to acetic acid (HBut/HAc) ranged from 1.13 to 2.05 (Fig. 4B). Without thermal pre-treatment, the HBut/HAc ratio was between 0.34 and 0.59. Heat shock pre-treatment reduced the accumulation of formic acid over all loading rates by 1.85 times. With the oven pre-treatment and 50 g/l substrate concentration, the greatest average butyric acid concentration was reached at 10.80 g/l followed by the microwave at 9.48 g/l. Microwave pre-treatment led to a single peak concentration of 24.01 g/l butyric acid at a substrate concentration of 50 g/l sucrose. At a substrate concentration of 100 g/l of sucrose and due to microwave pre-treatment, the butyric acid concentration increased 2.95 times up to 8.29 g/l. In contrast, the concentration of formic acid decreased to 13.14 g/l (1.93-fold). The amount of acetic acid was little influenced by the pre-treatment—in both the oven and the microwave experiments. Of the seven VFAs analysed, formic, acetic, and butyric acid were the most frequently represented (in total > 95%). In the untreated controls, 2.73 g/l butyric acid and 29.03 g/l formic acid were formed at the same substrate concentration of 50 g/l. None of the experiments contained larger amounts of propionic (maximum of 0.18 g/l) or lactic acid (maximum of 1 g/l). Significant concentrations of iso-butyric acid (0.02 g/l) and valeric acid (0.01 g/l) could not be determined in any of the tests. After an experimental period of seven days, average pH values of 3.71 (SD = 0.09) for the control and 4.16 (SD = 0.28) for heat-treated experiments were measured. At the start of the experiments, the pH value was 7.56 (SD = 0.13) for all scenarios and was not adjusted.

### The kinetics of hydrogen production after heat shock application

In the untreated experiments, gas production started immediately after adding the substrate (Figs. 4C, D, with 75 g/l sucrose as substrate). This was followed by a rapid accumulation of biogas over the next 7 h. After approximately 16 h, a noticeable plateau in gas production was reached, which came to an almost complete standstill after about 96 h. In contrast, total gas production in the heat shock experiments did not start until 6 h after feeding but then ascended steeply and continuously until the 36th hour. The subsequent rise is significantly less steep compared to the control experiment and nearly ceases entirely after 108 h. This pattern was observed with both pre-treatment, the oven and the microwave. The rate of gas formation in the heat-treated trials was less steep compared to the untreated control (Fig. 4C), yet the robust gas formation remained active for almost twice as long (30 h) compared to the control trials (16 h). The experiments demonstrated that heat shock pre-treatment leads to more consistent and prolonged gas production and significantly increases the total gas volume, albeit with a delay in gas production for the initial 6 hours.

Statistical significance was assessed using ANOVA (Fig. 4A) to better recognise whether the differences between the various treatments were significant. For example, at a substrate concentration of 100 g/l of sucrose, there is no significant difference between microwave- and oven-assisted pre-treatment regarding hydrogen volume, total gas volume, efficiency, or hydrogen percentage (Fig. 4A).

## Discussion

### Influence of substrate and feeding rate

Several research papers conclude that carbohydrate-rich substrates such as sugar, starch, or cellulose are especially effective for dark fermentation [42–44]. In addition to testing these suitable substrates, protein-rich and fat-containing substrates were also tested. The aim was to determine the suitability or unsuitability of these substrates in combination with heat shock pre-treatment of the entire initial sludge. Heat shock pre-treatment appeared to select for a microbial community less capable of metabolising bread, gelatine, and coconut oil, as evidenced by a decrease in gas production compared to the control. Research by [45–48] indicated that dark fermentation of gelatine and oily wastewater from the olive industry encounters challenges, resulting in similar limited hydrogen yields.

### Application of shorter heat shocks to high-loaded dark fermentation processes

One recent study states that inhibiting H_2_-consuming microorganisms, such as hydrogenotrophic methanogens, homoacetogens, lactic acid bacteria, propionate-producing bacteria, and sulphate reducers, is a crucial step in H_2_ production by dark fermentation in mixed microbial communities [49]. Many studies have used heat shocks to generate a small volume of enriched hydrogen-producing microbial community, which is then inoculated into a medium [50–52].

The study carried out here has demonstrated that the use of already fermented sewage sludge from anaerobic biogas production expands the usability of this waste stream in terms of cascade recycling and increasing its industrial relevance. This strategy allows for the selection of spore-forming hydrogen-producing microorganisms and the inhibition of non-spore-forming hydrogen-consuming microorganisms from heterogeneous microflora. Methanogens, which belong to the domain of archaea, are not able to form spores to survive extreme situations such as the applied heat shock. As several of them are hydrogenotrophic microorganisms, they consume the hydrogen produced to chemically reduce CO_2_ to methane. Through pre-treatment with heat shocks, methanogens can be inhibited to favour hydrogen production [53, 54]. Processes involving heterogeneous microflora are considered more suitable than pure cultures due to their simpler process control and efficient substrate conversion, provided that the medium undergoes pre-treatment [55]. Pineda-Muñoz et al. succeeded in generating high hydrogen yields by combining heat shock and ultrasonic treatment but did not completely suppress methane production despite nearly identical heat shock conditions [56]. Singhal and Singh investigated the effect of different microwave intensities on an inoculum [52]. In contrast to the study conducted here, they did not treat the entire sludge, but only a small inoculum of 20% of the total volume. Their focus was on the different levels of irradiation and not on the heat shock itself, which is why they carried out the treatment for only 5 min. They achieved a maximum hydrogen yield of 14 mmol H2/mol sucrose with microwave pretreatment at power levels from 160 to 800 W.

With the shorter microwave heat shocks (compared to conventional oven heat shocks) at a lower temperature, it was possible to completely inhibit methane production in the current experiments. Other researchers have also tried to inhibit methanogenesis by heat shocks. For example, Hasyim et al. conducted a shock at 105 °C for 20 min in an autoclave [57], while Wang et al. performed a 70 °C heat shock for 60 min with the support of free ammonia [51] to fully inhibit methane production. Although Hasyim et al. [57] and Wang et al. [51] were successful in inhibiting methanogens, the microwave has the advantage of heating the sludge medium 6.4 times faster than an oven. The experiments conducted here have demonstrated that even shorter and less energy-intensive heat shocks can completely eliminate methane production and thereby facilitate biological hydrogen production from sewage sludge. Notably, the experiments were performed in batches. In a continuous set-up, it is conceivable that methanogens would gradually adapt to the temperature shocks.

The recorded hydrogen formation rates are relatively low compared to more recent studies [58–60], but it is important to note that the present results were achieved despite heavy overfeeding. It must be considered that the current values correspond to the median of a minimum of six tests each. Compared to other works, the present results stand out due to the high number of replicates, which enabled a more robust and meaningful statistical analysis to evaluate the generated data. Considering the increased formation of butyric acid (Fig. 4B), a theoretical maximum hydrogen yield of 2 mol H_2_/mol hexose is possible [61]. With 1.09 and 1.01 mol H_2_/mol hexose in the present study, the achieved yield is much lower than the theoretical yield. Nevertheless, these results are in a similar range to Abdallah et al., who reached 1.1 mol H_2_/ mol hexose in a similar set-up [62]. As in the presented results, Abdallah et al. used no pH control, and they used lower substrate concentrations of 25 g/l.

Overall, the results indicate a high efficiency in the present experiments, even under high overfeeding concentrations and fast acidification due to missing pH control. In most studies, only triplicates were carried out and slightly higher hydrogen efficiencies were achieved. Compared to the high number of repeated experiments in the current study, other studies have a lower statistical certainty. Differences with other studies can arise due to the influence of the sludge used. It is possible that better results can be achieved with sewage sludge from other plants or with fermentation sludge from agricultural biogas plants. The composition of the sludge is often subject to seasonal fluctuations and therefore has a major influence on fermentation performance.

Hydrogen yield could potentially be increased further via pH controlling, since pH values of 4.0 to 4.5 harm hydrogen production [63]; this could explain the decrease in hydrogen formation as the substrate concentration increases. Increased overfeeding acidifies the reactor much faster and inhibits hydrogen formation. Gioannis et al. investigated the effect of pH on hydrogen formation. They discovered that the highest hydrogen production was attained at pH 6.0 [27]. In addition, the microbial community can be inhibited by undersaturation or supersaturation depending on the substrate concentration, as [64] have shown using chicken manure. In the present study, possible explanations for inhibition at high substrate concentrations might include inhibition due to metabolic products, increased osmotic pressure, or the substrate itself.

Compared to the untreated control (0.66 Nl/l) the hydrogen formation for the 75 g/l substrate concentration was enhanced six fold with microwave pre-treatment (3.97 Nl/l) and 7.3-fold with oven pre-treatment (4.79 Nl/l). These results are in accordance with those reported by Baghchehsaraee et al. [65] and O-Thong et al. [63], who reported a 5.1-fold (80 °C for 30 min) and 7.9-fold (100 °C for 1 h) increase as a result of thermal pre-treatments. The experiments carried out here yielded a high standard deviation even if the experimental conditions stayed the same. However, the standard deviation of the hydrogen efficiency could be slightly reduced in trials with 50 g/l sucrose feed and strongly reduced with 75 g/l sucrose feed by using the microwave for pre-treatment.

Among the tested conditions, microwave pre-treatment with a high substrate loading of 50 g/l sucrose resulted in the greatest hydrogen yield (1.01 mol H2/mol hexose) and butyric acid production (24.01 g/l). This concentration also resulted in the greatest total and relative median butyric acid production (microwave: 9.5 g/l, 31%; oven: 10.8 g/l, 32%) compared to all other tested concentrations. Additionally, hydrogen production efficiency was maximised at a substrate concentration of 50 g/l for both heat shock scenarios (microwave: 0.58 mol H_2_/ mol hex; oven: 0.86 mol H_2_/ mol hex). For microwave pretreatment, 100 g/l may also be economically feasible due to superior total hydrogen yields (5.47 Nl/l).

### Impact of the pretreatment on microbial community dynamics

The stability of AD systems is related to a higher diversity of species [66]. However, by exposing the microbial community to extreme conditions, a robust microbial community can be created. In this study, stable hydrogen production was achieved despite a decline in the diversity of species recorded and a strong dominance of *Clostridium *sensu* stricto 1*. The process and the microbial diversity were limited by the high production of organic acids and the resulting rapid drop in pH. The predictability of the microbial community was significantly higher and more consistent due to the microwave heat shock pre-treatment. Additionally, the growth of *Clostridium *sensu stricto* 1* was more dominant than in the oven-pretreated experiments. The strong shift in the microbial community towards *Clostridia* due to heat shock pre-treatment corresponds to the expectations according to the literature as they had higher relative abundances in thermophilic digesters and are known spore–formers [67]. Unfavourable conditions such as high temperatures make it difficult for non-spore-forming organisms to survive. As a result, almost exclusively the spore-forming *Bacillota* represented by *Clostridiaceae* grew explosively in the heat-treated experiments. Tang et al. observed a similar shift to *Clostridium sensu stricto* *1* in their experiments when the initial pH was increased from 4.0 to 11.0 [68]. In the present experiments the starting pH was at 7.5 and rapidly decreased to 4.0 to 4.5 after 48 h. *Clostridia* are well–known as potent hydrogen producers, as evidenced by numerous research studies conducted in recent years [69–72].

In the control experiments, the *Bacilli* were able to gain a growth advantage, as they were already more strongly represented in the initial sludge. Interestingly, the relative abundance of the class *Bacteroidia* remains unchanged in all experiments, hovering around 4%. In comparative studies, a high relative abundance of *Bacteroidetes* is often found in single-stage biogas processes [73, 74]. In the experiments of Chen et al. [75] the relative abundance of *Bacteroidetes* increased strongly with organic overload. *Bacteroidota*-coupled secondary fermentation processes normally carry out biological acid degradation. Both the control and the heat shock experiments revealed the same abundances of *Bacteroidota*, suggesting that these were inhibited primarily by the overfeeding rather than the heat shock.

In the microwave-pretreated experiments *Clostridium sensu stricto 1* demonstrated a strong dominance. This genus describes strictly anaerobic fermenting spore-formers [76]. During the metabolisation of sugars and proteins, the main fermentation products are butyric and acetic acid [77]. The genus is also able to produce lactic acid and ethanol, propanol or butanol. In the present experiments, members of *Clostridium sensu stricto 1* were able to produce large amounts of butyric acid even under severe overfeeding rates, demonstrating their robustness under extreme conditions. They likely benefited more from the high sucrose load. The population of the genus *Romboutsia*, which comprises around 13% of the microbial community in control experiments, remained almost unaffected by the heat shock pre-treatment, with slightly lower abundances around 7% in the heat-shocked experiments. According to Gerritsen et al. [78] *Romboutsia* is a potential acetogen capable of assimilating carbon via the Wood–Ljungdahl pathway (reductive acetyl-CoA pathway). The consistent relative abundance of *Romboutsia* could partly explain the high formic acid concentrations observed in all experiments (Fig. 4B). These bacteria primarily ferment sugars, converting them into acetate, formate, and lactate [79]. The control experiments indicated that the microbial community was dominated by *Streptococci* at the lower substrate concentration of 50 g/l sucrose. These are facultative anaerobes that ferment glucose under anaerobic conditions to produce lactic acid, acetic acid, propionic acid, and formic acid [80]. Only the untreated experiments showed an increase in propionic acid concentrations compared to the initial concentrations in the inoculation sludge. It is probable that the propionic acid formers in the sludge, like *Streptococcus*, lack mechanisms to survive high temperatures, unlike spore formers. It is not reported that *Streptococci* produce any gas; this suggests that the carbon dioxide in the untreated experiments was produced by the other microorganisms present. *Streptococci* growth was severely inhibited by heat shock pre-treatment. Small populations of the spore-forming *Paeniclostridium* [81] were only present in the heat-treated experiments with substrate concentration of 50 g/l, while larger amounts were found in the untreated control experiments. The results suggest that the class did not benefit from the heat shock treatment, but from the overfeeding.

### Bio-based production of short-chain organic acids

Large quantities of acids were formed in both the untreated and the heat-shock-treated experiments. In combination with the low pH value at the end of the test series and the results of the microbial community analysis, it can be assumed that no effective acid degradation takes place in any of the experiments and that there is instead a strong accumulation of acids. While total acid concentration remains very similar regardless of the treatment and substrate concentration (except for the control with 75 g/l of sucrose), the proportion of individual acids changes as a result of the heat shock pre-treatment.

The butyric acid to acetic acid molar ratio curves illustrate that the heat shock initialised the production of less acetic acid, while the amount of butyric acid increased 2.7 times. The high HBut/HAc molar ratios for all heat-shocked experiments (Fig. 4B) indicate that hydrogen was produced via the butyrate pathway [56]. Without heat pre-treatment, the HBut/HAc ratio indicates that hydrogen was formed via the acetate pathway.

While in experiments with microwave treatment the butyric acid concentration increased with heavy overfeeding (100 g/l), it decreased with increasing loading in experiments with oven pre-treatment. The observed high formic acid concentrations might be associated with the presence of formic acid-producing bacteria like *Romboutsia* [79] and *Streptococci* [80]. This aligns with findings reported by [82], who observed a link between elevated hydrogen and carbon dioxide partial pressures and increased formate production in their studies. Given the high hydrogen and carbon dioxide concentrations and the substantial gas quantities observed in the present experiments, it is reasonable to assume that the reactor system had high hydrogen and carbon dioxide partial pressures. Per previous findings by [83], formate synthesis may compete with certain hydrogen-producing reactions. [84] and [85] demonstrated experimentally that some clostridial strains can utilise formate as an additional substrate. This phenomenon could explain the high hydrogen and carbon dioxide yields observed in the presented *Clostridia*-driven fermentations. Heat pre-treatment reduced the variety of acids as no propionic acid was formed anymore. Both lactic and propionic acid had very low concentrations, corresponding with the results of Fang and Liu [86]. Their formation is typical for processes with high substrate concentrations [27, 87], and it is therefore suggested that only a few propionic acid formers were already present in the seed sludge and that the conditions tested did not favour their growth. The experiments demonstrated the feasibility of producing high concentrations of bio-based butyric acid through dark fermentation processes by combining microwave heat shocks and heavy overfeeding. To increase the formation capacity of organic acids, it is recommended to improve buffering capacity, control pH value, and constantly remove organic acids. According to Pineda-Muñoz et al., decreasing pH leads to increased formation of acetic and formic acid [56]. Therefore, regulating pH could result in an even higher ratio of butyric acid.

### Industrial relevance

As the demand for green hydrogen intensifies [88], biological hydrogen production via dark fermentation is gaining significant traction due to its environmentally friendly nature. However, for fermentative hydrogen production to become a commercially viable alternative to current methods, achieving economic feasibility is crucial. The investigations carried out in this study indicate that one litre of heat-treated sludge can yield an average of 5.92 m^3^ of hydrogen per m^3^ sludge at a substrate concentration of 50 g/l. Subtracting the average values for the control from this, the heat treatment results in an additional gain of 5.02 m^3^ hydrogen per m^3^ of sludge. The thermal energy required to heat the seed sludge from 38 °C to 80 °C is 49.04 kWh/m^3^, given that it takes 4.2 kJ to increase the temperature of 1 L sludge by 1 °C. The additional energy generated in the form of hydrogen due to the heat shocks corresponds to 54.18 kJ/l sludge, which corresponds to 15.06 kWh/m^3^ sludge. Based on the 49.04 kWh/m^3^ for the heat shock, the energy loss for the heat shock process is 33.98 kWh/m^3^. This amount of energy must at least be recovered using a heat pump to make this concept energetically attractive.

Approved high-temperature heat pumps, which are currently available on the market, can achieve a coefficient of performance (COP) of 4.3 when heating to 80 °C from a starting temperature of 20 °C (per data from ENGIE Refrigeration GmbH in [89]). This implies that 1 kWh of electrical energy can produce up to 4.3 kWh of thermal energy. To generate 49.04 kWh of thermal energy, a heat shock requiring 11.4 kWh of electricity would be needed, assuming a coefficient of performance (COP) of 4.3. At this COP, the heat shock generates an additional energy output of 3.66 kWh/m^3^ of sludge compared to the calorific energy of the additional hydrogen. The process would also be energetically profitable due to further energy losses during the heat exchange process with the sludge, which are not considered here. Future technological improvements that increase the COP could make enhanced hydrogen production through heat shocks even more sustainable.

In view of the different costs of hydrogen and electric energy, the economic appeal of the concept could be retained even if the hydrogen equivalent of one kWh is priced higher than electricity itself. Based on Machhammer et al. and Bukold, a good benchmark for the costs of hydrogen production is the average price for steam reforming of 1.70 €/kg H_2_ [90, 91]. The energy cost for a heat shock using a high-temperature heat pump with a COP of 4.3 would be 0.4 €/m^3^ of sludge, (based on an average industrial electricity price of 35 €/MWh). Selling the produced hydrogen at the same price of 1.70 €/kg would generate an average revenue of 0.91 € based on the amount of hydrogen from one cubic metre of heat-shocked sludge. This corresponds to a profit of 0.51 €/m^3^ of sludge. Despite a net energy loss, the production of hydrogen through heat shocks proves to be more cost-effective compared to other alternative methods of hydrogen production. The current costs of hydrogen production without steam reforming are quantified by Gerloff at 5.20 €/kg [92]. Lowering the heat shock temperature could significantly decrease the energy requirement and thus the essential costs while potentially yielding similar or even higher hydrogen yields [65]. These calculations are based on ideal assumptions and the successful scaling of trials in an industrial setting, with a focus on electricity costs. Studies by [93] and [94] report similar findings and support the economic potential of dark fermentation for hydrogen production. However, these studies lack a direct cost analysis of the heat-shock process and a comparison with the non-heat-shocked scenario.

## Conclusion

Heat shocks are often applied to trigger hydrogen formation in mixed-culture fermentation. Based on the present study, it can be concluded that particularly short heat shock treatments with maximum exposure times of 10 min, and temperatures of 80 °C are feasible if microwaves are applied instead of a conventional oven. Often, only small volumes are heat-treated as inoculum in comparable works. The present work suggests that the entire reactor content should be treated to reach industrial relevance. At the optimal substrate concentration of 50 g/l of sucrose, this yields up to 1.01 mol H_2_/mol hexose and 24.01 g/l of butyric acid. To make the concept more reliable, future work might assess the possibility of reducing the high standard deviation in both hydrogen and volatile fatty acid yields. Since *Clostridia* dominated in heat-treated reactors (up to 83%), future attempts could adjust the reaction conditions to better meet the nutrient requirements of *Clostridia* specifically. Furthermore, easily digestible carbohydrates should be preferred substrates. To make complex mixtures of organic waste more accessible for the presented method, further research might investigate new mechanisms to enable hydrogen production from proteins and lipids.

Calculations based on the present results indicate that state-of-the-art heat pumps might enable an energetically sustainable upscaling that is economically competitive with electrolysis for hydrogen production. In future work, excess heat from the heat shocks could be used to heat a second stage for anaerobic methane formation, which would make the concept even more sustainable.

## Supplementary Information

Supplementary Material 1. Figure S1: Microbial community on genus level as relative abundances differentiated according to pre-treatment (substrate concentrations of 50 and 100 g/l). Only the microorganisms with an abundance < 5 % are presented. Others with abundances below 0.5 % are summarised as others < 0.5 %.

Supplementary Material 2. Figure S2: Microbial community on genus level as relative abundances differentiated according to pre-treatment. The substrate concentration was 100 g/l sucrose. (A) Only the most abundant microorganisms with an abundance > 5% are presented. Others with abundances below 5 % are summarised as others < 5.0 %. (B) Breakdown of minor microorganisms below 5 % abundance. Organisms with abundances below 0.5 % are summarised as others < 0.5 %.

Supplementary Material 3. Figure S3: Microbial community on phylum level as relative abundances differentiated according to pre-treatment (substrate concentrations of 50 and 100 g/l). Only microorganism communities with abundances larger than 2 % are presented. Other phyla are summarised as “others < 2 %”.

## Data Availability

Raw data from sequencing of 16S rRNA gene amplicon high-throughput sequences are available at GenBank (project ID PRJNA1150417) and can be accessed with the following link: http://www.ncbi.nlm.nih.gov/bioproject/1150417.
