# Supplementary figures and images for: Microwave-assisted organic acids and green hydrogen production during mixed culture fermentation
Source: Biotechnol Biofuels Bioprod. 2024 Sep 28;17:123. doi: 10.1186/s13068-024-02573-7 (PMC11439308; doi:10.1186/s13068-024-02573-7)

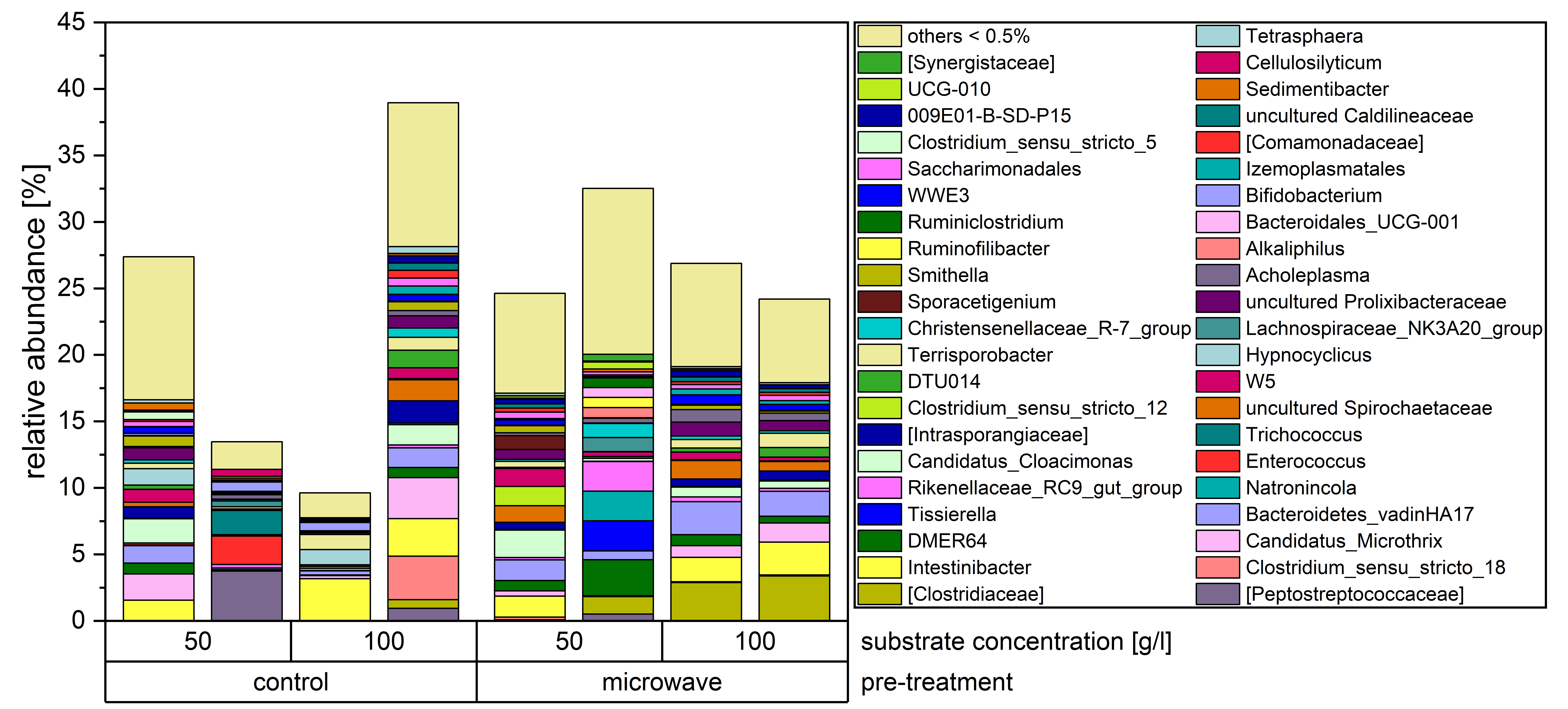

Supplement: Supplementary file 1 — Supplementary Material 1. Figure S1: Microbial community on genus level as relative abundances differentiated according to pre-treatment (substrate concentrations of 50 and 100 g/l). Only the microorganisms with an abundance < 5 % are presented. Others with abundances below 0.5 % are summarised as others < 0.5 %. [file 13068_2024_2573_MOESM1_ESM.tif]

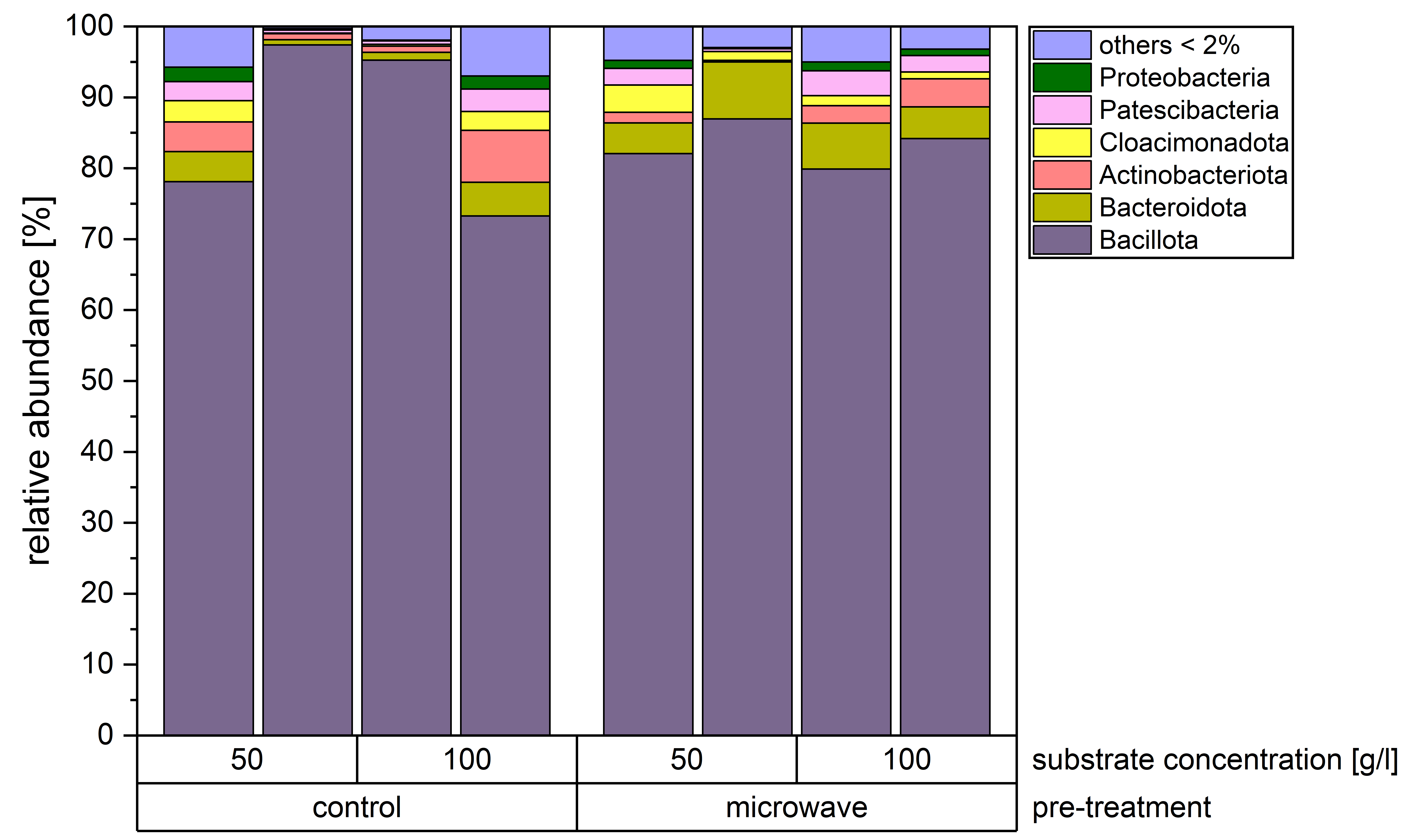

Supplement: Supplementary file 3 — Supplementary Material 3. Figure S3: Microbial community on phylum level as relative abundances differentiated according to pre-treatment (substrate concentrations of 50 and 100 g/l). Only microorganism communities with abundances larger than 2 % are presented. Other phyla are summarised as “others < 2 %”. [file 13068_2024_2573_MOESM3_ESM.tif]
